# Initial Glutathione Depletion During Short-Term Bed Rest: Pinpointing Synthesis and Degradation Checkpoints in the γ-Glutamyl Cycle

**DOI:** 10.3390/antiox13121430

**Published:** 2024-11-21

**Authors:** Filippo Giorgio Di Girolamo, Filippo Mearelli, Mariella Sturma, Nicola Fiotti, Kaja Teraž, Alja Ivetac, Alessio Nunnari, Pierandrea Vinci, Boštjan Šimunič, Rado Pišot, Gianni Biolo

**Affiliations:** 1 Department of Medical Surgical and Health Sciences, ASUGI, University of Trieste, 34127 Trieste, Italy; fgdigirolamo@units.it (F.G.D.G.); sturma@units.it (M.S.); fiotti@units.it (N.F.); kaja.teraz@zrs-kp.si (K.T.); alya.ivetac@gmail.com (A.I.); 2 Hospital Pharmacy, Cattinara Hospital, Azienda Sanitaria Universitaria Giuliano Isontina, 34148 Trieste, Italy; 3 Clinica Medica, Department of Medical Surgical and Health Sciences, ASUGI, University of Trieste, 34127 Trieste, Italy; filippo.mearelli@asugi.sanita.fvg.it (F.M.); alessionunnari@gmail.com (A.N.); pierandrea.vinci@asugi.sanita.fvg.it (P.V.); 4 Institute for Kinesiology Research, Science and Research Centre Koper, 6000 Koper, Slovenia; bostjan.simunic@zrs-kp.si (B.Š.); rado.pisot@zrs-kp.si (R.P.)

**Keywords:** antioxidant, muscle unloading, glutathione turnover, γ-glutamyl cycle

## Abstract

Hypokinesia triggers oxidative stress and accelerates the turnover of the glutathione system via the γ-glutamyl cycle. Our study aimed to identify the regulatory checkpoints controlling intracellular glutathione levels. We measured the intermediate substrates of the γ-glutamyl cycle in erythrocytes from 19 healthy young male volunteers before and during a 10-day experimental bed rest. Additionally, we tracked changes in glutathione levels and specific metabolite ratios up to 21 days of bed rest. Using gas chromatography-mass spectrometry and the internal standard technique, we observed a 9 ± 9% decrease in glutathione levels during the first 5 days of bed rest, followed by an 11 ± 9% increase from the 5th to the 10th day, nearly returning to baseline ambulatory levels. The cysteinyl-glycine-to-glutathione ratio, reflecting γ-glutamyl cyclotransferase activity (a key enzyme in glutathione breakdown), rose by 14 ± 22% in the first 5 days and then fell by 10 ± 14% over the subsequent 5 days, again approaching baseline levels. Additionally, the γ-glutamyl cysteine-to-cysteine ratio, indicative of γ-glutamyl cysteine synthetase activity (crucial for glutathione synthesis), increased by 12 ± 30% on day 5 and by 29 ± 41% on day 10 of bed rest. The results observed on day 21 of bed rest confirm those seen on day 10. By calculating the ratio of product concentration to precursor concentration, we assessed the efficiency of these key enzymes in glutathione turnover. These results were corroborated by directly measuring glutathione synthesis and degradation rates in vivo using stable isotope techniques. Our findings reveal significant changes in glutathione kinetics during the initial days of bed rest and identify potential therapeutic targets for maintaining glutathione levels.

## 1. Introduction

Muscle unloading leads to the production of reactive oxygen and nitrogen species (RONS), which may play a role in triggering metabolic pathways that contribute to inactivity-induced muscle atrophy [1,2,3], insulin resistance [4], and inflammation [5]. The generation of RONS also activates antioxidant defenses and elevates biomarkers of oxidative stress. Studies by our group and others have demonstrated that systemic [6] and muscle-specific [7,8,9,10] oxidative stress biomarkers are activated in the early stages of experimental bed rest (that is, within 5 days of bed rest), even before muscle wasting begins. Our research found that after about one month of bed rest, a reduction in vastus lateralis muscle thickness was inversely correlated with an increase in protein carbonylation, an oxidative stress marker, in the same muscle [11,12]. These changes were accompanied by an increase in the synthesis rate of muscle glutathione, a key antioxidant [11]. After the first month of bed rest, the intensity of oxidative stress diminishes, and some biomarkers return to baseline levels [13]. Similarly, the progression of muscle atrophy slows down after one month [14]. This suggests a possible link between the timing of oxidative stress and muscle atrophy during bed rest, with a potential cause–effect relationship. Therefore, studying the initial phase of bed rest is crucial to understanding the mechanisms that activate oxidative stress and their potential connection to physiological changes induced by mechanical muscle unloading.

**Figure 1:**
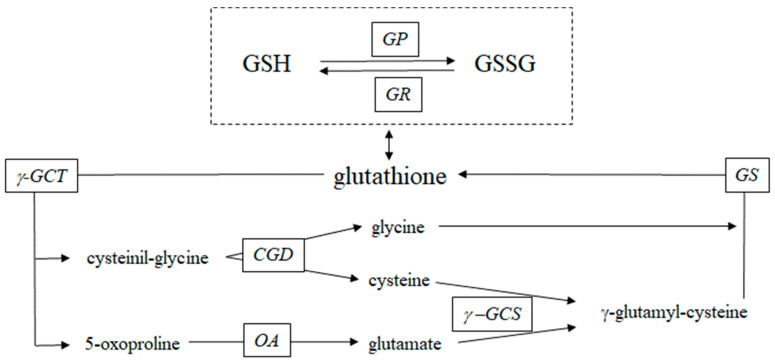
The γ-glutamyl cycle. This schematic illustrates the key enzymes and intermediates involved in glutathione metabolism within erythrocytes. GSH (reduced glutathione): the primary antioxidant form of glutathione, which can be oxidized to GSSG (oxidized glutathione) to maintain redox balance; GP (glutathione peroxidase): catalyzes the oxidation of GSH to GSSG, neutralizing reactive oxygen species; GR (glutathione reductase): converts GSSG back to GSH, thus regenerating the antioxidant pool; γ-GCT (γ-glutamyl cyclotransferase): degrades glutathione into cysteinyl-glycine and 5-oxoproline, a crucial step in the breakdown process; CGD (cysteinyl-glycine dipeptidase): further hydrolyzes cysteinyl-glycine to yield free cysteine and glycine; OA (5-oxoprolinase): converts 5-oxoproline into glutamate, aiding in the recycling of amino acids; γ-GCS (γ-glutamyl cysteine synthetase): catalyzes the formation of γ-glutamyl-cysteine from glutamate and cysteine, the rate-limiting step in glutathione synthesis; GS (glutathione synthetase): combines γ-glutamyl-cysteine with glycine to form glutathione. These enzymes work in concert to maintain glutathione levels and antioxidant capacity within the cytoplasm of erythrocytes, protecting against oxidative damage.

The glutathione system is a major intracellular antioxidant and detoxifying agent [15]. Glutathione is a tripeptide composed of cysteine, glutamic acid, and glycine, which exerts its antioxidant function by converting from its reduced form (GSH) to its oxidized form (GSSG) via the enzyme glutathione peroxidase. The antioxidant capacity of glutathione depends on its total concentration, predominantly in the reduced form, and the ratio of the reduced to oxidized forms, which reflects the redox state. The oxidized form constitutes less than 1% of the reduced form. Glutathione is degraded in the cell cytoplasm through the glutathione cycle, also known as the γ-glutamyl cycle, resulting in the release of free amino acids—glutamate, cysteine, and glycine—and its re-synthesis from these same precursors. This cycle was first proposed by Meister et al. [16,17] and has since been further refined [18,19,20]. The glutathione cycle involves two key enzymatic steps in its non-ribosomal biosynthesis in the cytosol [21]. The first step, catalyzed by γ-glutamylcysteine synthetase (or glutamate cysteine ligase), combines glutamate and cysteine in an ATP-dependent reaction to form γ-glutamylcysteine. In the second step, glutathione synthetase adds glycine to γ-glutamylcysteine, again using ATP, to produce glutathione (γ-glutamylcysteinylglycine). The main factors influencing glutathione synthesis are the availability of cysteine, the sulfur-containing amino acid precursor, and the activity of the rate-limiting enzyme γ-glutamylcysteine synthetase, which consists of catalytic and modifier subunits [21,22,23]. Once synthesized, glutathione can be broken down by the cytoplasmic ChaC family of γ-glutamyl cyclotransferases, which specifically target glutathione for degradation [24,25,26]. This degradation releases 5-oxoproline and cysteinylglycine, which are further processed into glutamate, cysteine, and glycine, respectively. These amino acids are then reused to synthesize new glutathione molecules, maintaining the cycle’s efficiency in cellular antioxidant defense and detoxification [27]. Together, these metabolic pathways form the glutathione or γ-glutamyl cycle, a recycling mechanism that sustains intracellular levels of this crucial tripeptide [28]. Variations in glutathione levels are the result of changes in the synthesis and degradation rates of the tripeptide (Figure 1).

Glutathione metabolism is essential for maintaining cellular redox balance and is tightly regulated across various tissues and cellular compartments. Synthesis of glutathione predominantly occurs in the liver [20,21], which is the main site for systemic production, but it also takes place in other tissues, including the kidneys, lungs, brain, gut, skeletal muscle, heart and erythrocytes [16,21,29]. Within cells, glutathione is primarily found in the cytoplasm, but significant amounts are also present in mitochondria and the nucleus, where it mitigates oxidative damage and supports cell function [30]. In addition to its intracellular roles, glutathione circulates extracellularly in the plasma, providing systemic antioxidant defense by balancing redox states in the blood [31]. The degradation of glutathione is largely facilitated by the γ-glutamyl cycle. Here, γ-glutamyl cyclotransferase initiates glutathione breakdown, generating metabolites that allow for the recycling of constituent amino acids—glutamate, cysteine, and glycine [17,32].

Glutathione is found in higher concentrations in erythrocytes [33], where it offers both local and systemic antioxidant protection [29,31,34]. Glutathione plays a key role in protecting hemoglobin, red blood cell enzymes, and cell membranes from oxidative damage [25,35]. Degradation is driven by cytoplasmic γ-glutamyl cyclotransferase, which converts glutathione into intermediates that can be recycled back into the tripeptide. Erythrocytes depend on this pathway to protect against oxidative stress, as they are continually exposed to reactive oxygen species in the bloodstream. Consequently, the erythrocyte-specific pathway of glutathione metabolism serves as a critical antioxidant mechanism, sustaining cellular defense and reflecting systemic redox changes in response to various physiological stresses, such as those induced by bed rest [6,11,36,37,38,39,40,41,42,43].

In this study, we assessed the glutathione system and all intermediate metabolites of the γ-glutamyl cycle during short-term bed rest in the erythrocytes of healthy volunteers. Analyses were conducted on the 5th and 10th days of bed rest, with enzymatic activities determined by expressing the ratio between product and precursor. The objective was to correlate changes in glutathione concentrations with variations in the activity of key enzymes regulating the synthesis and degradation of the tripeptide. Additionally, to validate this new approach, we measured red blood cell concentrations of γ-glutamyl cycle intermediates in blood samples from two previously published studies where glutathione turnover was measured [44,45]. In one study [45], the administration of an alkalizing agent during bed rest led to increased glutathione concentrations and reduced turnover, indicating inhibited degradation. In the other study [44], prolonged hypoxia during bed rest resulted in increased glutathione concentrations and increased turnover, indicating stimulated synthesis.

## 2. Materials and Methods

### 2.1. Subjects and Experimental Design

The studies were part of the Italian Space Agency (ASI) project “MARS-PRE Bed Rest SBI 2019” and the European Union Italy-Slovenia Interreg project “X-BRAIN-net”. Both bed rest studies were conducted at Izola General Hospital, Slovenia, in 2019 and 2023. The study protocols received approval from the National Ethical Committee of the Slovenian Ministry of Health (reference numbers: 0120-304/2019/9 and 0120-123/2023/9) and were carried out in compliance with the standards set by the Declaration of Helsinki. Before participation, all subjects were thoroughly informed about the study’s objectives, procedures, and potential risks, and written consent was obtained. Nineteen healthy, recreationally active young men (age: 23 ± 4 years; height: 1.82 ± 0.05 m; body mass: 78.8 ± 8.0 kg; body mass index (BMI): 23.8 ± 2.4 kg/m^2^) participated in the studies—10 in 2019 and 9 in 2023. Participants underwent medical screening prior to the study. Exclusion criteria included regular smoking, habitual drug use, blood clotting disorders, history of deep vein thrombosis, neuromuscular, metabolic, and cardiovascular conditions, past embolism, inflammatory diseases, psychiatric disorders, epilepsy, and the presence of ferromagnetic implants. Subjects were subjected to strict horizontal bed rest for 10 days in 2019 and 21 days in 2023, with no deviations from the lying position, muscle stretching, or static contractions allowed. Participants were continuously monitored via closed-circuit television and supervised by researchers and medical staff. They arrived at the hospital three days prior to bed rest for pre-bed rest measurements. During the study, subjects consumed an individually controlled, standardized diet (60% carbohydrates, 25% fats, and 15% proteins) and were allowed to drink water ad libitum. Blood samples were collected from each participant’s medial cubital vein in a postabsorptive state at 07:00 on the day before bed rest and on the 5th and 10th days of bed rest in the 2019 study, and on the 5th, 9th, and 21st days in the 2023 study. The MARS-PRE 2019 bed rest study design has been previously detailed [46]. Hematocrit levels were assessed using standard methods for all blood samples. Body composition, specifically fat-free mass (FFM) and fat mass (FM) was measured using multifrequency bioimpedance (BIA-Human Implus–DSmedica, Milan, Italy) both before and after the bed rest periods. Bioelectrical impedance measurements were taken in the morning after an overnight fast, with participants lying in bed for 30 min before the assessment to ensure proper body fluid redistribution.

### 2.2. Analyses

We measured erythrocyte concentrations of total glutathione (i.e., the combined levels of GSH and GSSG), dipeptide intermediates of the γ-glutamyl cycle (cysteinyl-glycine, and γ-glutamyl cysteine), and select amino acids (5-oxoproline, glutamic acid, glutamine, cysteine, glycine, and alanine) using gas chromatography-mass spectrometry (GC-MS) (HP 5890; Agilent Technologies, Santa Clara, CA, USA). These were analyzed as *N*-(t-butyldimethylsilyl)-*N*-methyltrifluoroacetamide derivatives, following established protocols [11,36,44,45]. Briefly, known quantities of internal standards—including [glycine-^13^C_2_,^15^N] glutathione, L-[^15^N] glycine, L-[3,3-^2^H_2_] cysteine, and L-[^15^N]alanine (Cambridge Isotope Laboratories, Andover, MA, USA)—were added to 200 μL of erythrocyte. The samples were processed as described previously [36], with dithiothreitol added to reduce any dimerized glutathione. The derivatives were measured using electron-impact ionization with selective ion monitoring at nominal *m*/*z* values of 366/363 for glutathione, 219/218 for glycine, 433/432 for glutamate, 432/431 for glutamine, 408/406 for cysteine, and 159/158 for alanine. The internal standards L-[^15^N] glycine, L-[3,3-^2^H_2_] cysteine, and L-[^15^N] alanine were also utilized to determine dipeptide concentrations. The concentration of γ-glutamyl-alanine was calculated using a standard curve between γ-glutamyl-alanine and L-[^15^N] alanine at a nominal *m*/*z* of 159/132. Similarly, the concentration of cysteinyl-glycine was determined using a standard curve between cysteinyl-glycine and L-[^15^N] glycine at a nominal *m*/*z* of 219/304, and γ-glutamyl-cysteine concentration was calculated by a standard curve between γ-glutamyl-cysteine and L-[3,3-^2^H_2_] cysteine at a nominal *m*/*z* of 408/306. Hematocrit levels were assessed using standard methods. The GSH/GSSG ratio in red blood cells was measured using a commercially available kit (Oxford Biomedical Research Inc., Rochester Hills, MI, USA). Erythrocyte concentrations of 5-oxoproline, glutamine, and glutamate were determined by adding known amounts of L-[^15^N] glutamate and L-[^15^N] glutamine as internal standards to the known volume of erythrocyte. Silylated derivatives were measured using electron-impact ionization with selective ion monitoring at nominal *m*/*z* values of 432/433 for glutamic acid, 431/432 for glutamine, and 300 for the 5-oxoproline derivative. The 5-oxoproline concentration was calculated using a standard curve between 5-oxoproline and glutamate at a nominal *m*/*z* of 300/432 [47]. Plasma levels of insulin, glucose, high-sensitivity CRP, total cholesterol, HDL-C, LDL-C, triglycerides, AST, ALT, albumin, and creatinine were measured by a certified external laboratory (Synlab Italia Srl, Brescia, Italy) using standard methods. The Homeostatic Model Assessment was applied to calculate insulin resistance (HOMA-IR) and insulin secretion (HOMA-β%) by standard equations [44] as follows: HOMA-IR = [fasting glucose (mg/dL) × fasting insulin (μU/mL)/405]; HOMA-β% = [360 × fasting insulin (μU/mL)]/[fasting glucose (mg/dL) − 63]. The expression of the catalytic and modulator subunits of γ-glutamyl cysteine synthetase in erythrocytes was measured via Western blot analysis in the 10 participants from the MARS-PRE Bed Rest SBI 2019 study. Protein concentrations of these enzyme subunits were expressed as a ratio to glyceraldehyde-3-phosphate dehydrogenase (GAPDH) protein levels, following previously established protocols [36]. The activities of the five γ-glutamyl cycle enzymes (Figure 1) were assessed by calculating product-to-precursor ratios (expressed as fraction or percent), including: γ-glutamyl cyclotransferase (cysteinyl-glycine-to-glutathione % ratio and 5-oxoproline-to-glutathione % ratio), cysteinyl-glycine dipeptidase (glycine-to-cysteinyl-glycine ratio and cysteine-to-cysteinyl-glycine ratio), 5-oxoprolinase (glutamate-to-5-oxoproline ratio), γ-glutamyl cysteine synthetase (γ-glutamyl-cysteine-to-cysteine % ratio, γ-glutamyl-cysteine-to-glutamate % ratio), and glutathione synthetase (glutathione-to-γ-glutamyl-cysteine ratio and glutathione-to-glycine ratio).

### 2.3. Validation of Glutathione Kinetics

We compared glutathione kinetics, as measured by intermediate levels in the erythrocyte γ-glutamyl cycle, with kinetics assessed by the rate of L-[^2^H_2_-glycine] incorporation into the tripeptide. This comparison was drawn from previous bed rest studies conducted under different experimental conditions, such as alkali supplementation [45] and hypoxia [44]. While the experimental design and results of glutathione kinetics using stable isotopes have been published earlier, the analysis of intermediate levels in the erythrocyte γ-glutamyl cycle was conducted later on blood samples that had been stored at −80 °C, as described above.

### 2.4. Statistical Analysis

All values are presented as means ± SD. The data were analyzed using repeated measures analysis of covariance (RM-ANCOVA), with time points (baseline, 5-day bed rest, and 10-day bed rest—combining results from days 9 and 10) as within-subject factors. Baseline values were used as covariates when applicable. Post hoc analysis was conducted, when appropriate (i.e., significant effects of bed rest), using *t*-tests with Bonferroni adjustment to evaluate the specific effects of bed rest days. We assessed the time course of bed rest-induced percentage changes in glutathione levels, the cysteinyl-glycine-to-glutathione ratio, and the γ-glutamyl-cysteine-to-cysteine ratio on the 5th, 10th, and 21st days of bed rest. Data from the MARS-PRE study (assessments on the 5th and 10th days of bed rest, *n* = 10) and the X-BRAIN-net study (assessments on the 5th, 10th, and 21st days of bed rest, *n* = 9) were combined with data from previous bed rest studies, assessments on the 10th day of bed rest, *n* = 11; see Ref. [44], assessments on the 21st day of bed rest, *n* = 7, (see Ref. [45]). Percentage changes at different time points were compared using paired or unpaired *t*-tests, as appropriate, with Bonferroni adjustments. All comparisons were considered statistically significant at *p* ≤ 0.05. Relationships between variables were analyzed using bivariate correlation with Pearson’s coefficient. Statistical analysis was performed using SPSS software (version 12; SPSS Inc., Chicago, IL, USA).

## 3. Results

FFM, an indicator of lean body mass, decreased from 62.4 ± 1.9 to 61.0 ± 1.7 kg (*p* = 0.002) after 10 days of bed rest in the 2019 study. FFM decreased from 66.5 ± 5.3 to 62.9 ± 4.5 kg (*p* = 0.001) after 21 days of bed rest in the 2023 study. FM, an indicator of adipose tissue and energy balance, did not change significantly from baseline (*p* = 0.75) after 10 days of bed rest (15.1 ± 6.1 and 15.0 ± 6.2 kg, respectively) in the 2019 study. FM increased significantly from baseline (*p* < 0.001) after 21 days of bed rest from 12.5 ± 4.7 to 15.6 ± 5.9 kg in the 2023 study.

Table 1 shows bed rest-induced changes in routine biochemical parameters in the postabsorptive state. During bed rest, plasma fasting insulin concentration increased significantly, indicating the development of inactivity-induced insulin resistance. Consistently, HDL cholesterol decreased, and the ratio of triglycerides to HDL cholesterol increased. The HOMA Insulin Resistance index tended to increase, whereas the HOMA Insulin Secretion index increased significantly. Total and LDL cholesterol significantly decreased during bed rest. Plasma liver enzymes, CRP, and creatinine did not change significantly.

Table 2 shows the levels of total glutathione and intermediate substrates of the γ-glutamyl cycle in erythrocytes in ambulatory conditions and during early and late bed rest. The reduced-to-oxidized glutathione ratio (GSH/GSSG) is also presented. Total glutathione decreased by −9 ± 9% during the first 5 days of bed rest and increased from day 5 to day 9 or 10 by 11 ± 9%, returning approximately to baseline ambulatory values. The ratio between oxidized and reduced glutathione did not change significantly during bed rest.

Cysteinyl-glycine and 5-oxoproline are direct products of glutathione catabolism; their intracellular concentration is about 0.5% and 9% of that of glutathione, respectively. γ-glutamyl cysteine is the direct precursor of glutathione synthesis, and its concentration is about 0.14% of that of glutathione. We did not observe any significant effect of bed rest on the concentration of any of the intermediate metabolites of the γ-glutamyl cycle. In contrast, bed rest significantly decreased cytoplasmic concentration of cysteine, the precursor of γ-glutamyl cysteine, together with glutamic acid.

Table 3 shows γ-glutamyl cycle enzyme activities estimated as product-to-precursor ratios or percent. γ-glutamyl cyclotransferase activity, estimated as cysteinyl-glycine/Glutathione, index of glutathione catabolism, significantly increased by 14 ± 22% during the first 5 days of bed rest and decreased from day 5 to day 9 or 10 by −10 ± 14%, returning approximately to baseline ambulatory values. Bed rest-related changes of cysteinyl-glycine/glutathione (i.e., γ-glutamyl cyclotransferase activity) and of total glutathione concentration from ambulatory to day 5 (Spearman R = −0.80) and from day 5 to day 10 (Spearman R = −0.53) were inversely correlated (*p* ≤ 0.01). The activity of γ-glutamyl cysteine synthetase, the key enzyme for glutathione synthesis, is expressed either as the ratio between γ-glutamyl cysteine to cysteine or as the ratio between γ-glutamyl cysteine to glutamate, significantly increased during bed rest. The ratio between γ-glutamyl cysteine to cysteine increased from basal by 12 ± 30% and 20 ± 41% at day 5 and at day 10, respectively. The ratio between γ-glutamyl cysteine to glutamate increased from basal by 14 ± 43% and 10 ± 35% at day 5 and at day 10, respectively. 5-oxoprolinase activity, expressed as glutamate-to-5-oxoproline ratio, significantly increased following bed rest.

Table 4 shows bed rest effects on expression levels of the γ-glutamyl cysteine synthetase enzyme in erythrocytes. There was a significant bed rest effect on γ-glutamyl cysteine synthetase—modulator subunit, while no bed rest effect was observed on γ-glutamyl cysteine synthetase—catalytic subunit.

**Figure 2:**
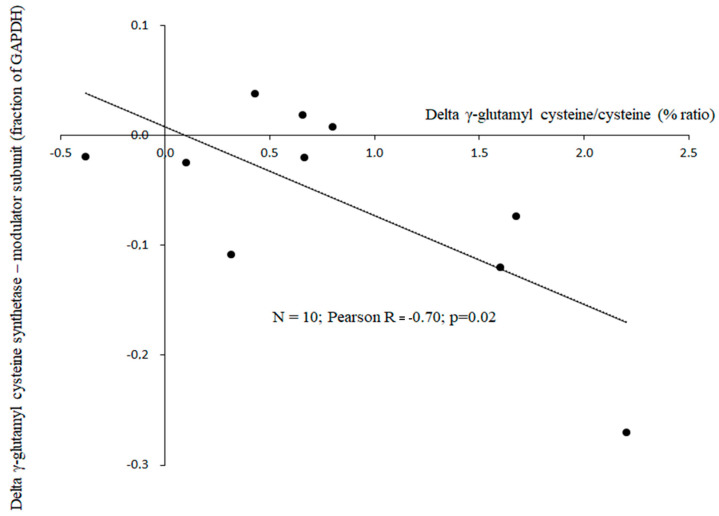
Relationship between bed rest mediated changes of the ratio between γ-glutamyl cysteine to cysteine and expression level of γ-glutamyl cysteine synthetase–modulator subunit from baseline to day 5 of bed rest in erythrocytes. The ratio between γ-glutamyl cysteine to cysteine gas was determined by chromatography-mass spectrometry and stable isotopes as internal standard. The expression level of γ-glutamyl cysteine synthetase–modulator subunit was determined by Western blot.

Figure 2 illustrates an inverse relationship (Pearson R = −0.70, *p* = 0.02, *n* = 10) between bed rest-induced changes in the γ-glutamyl cysteine-to-cysteine ratio and the expression level of the γ-glutamyl cysteine synthetase modulator subunit from baseline to day 5 of bed rest.

**Figure 3:**
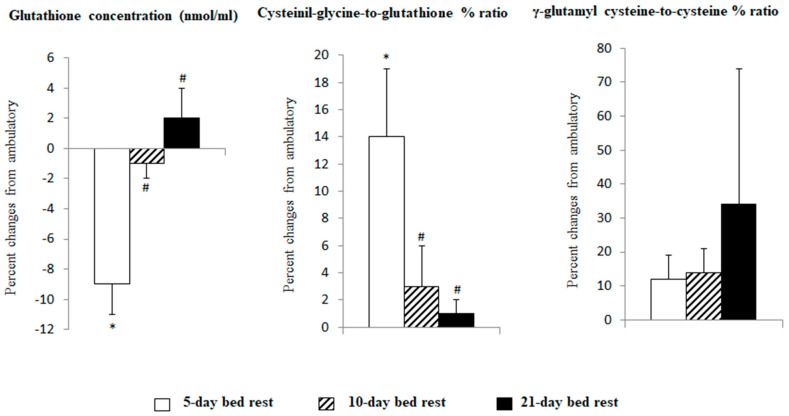
Changes from baseline of glutathione concentration, cysteinyl glycine-to-glutathione ratio (index of γ-glutamyl cyclotransferase activity a marker of glutathione degradation), and γ-glutamyl cysteine-to-cysteine ratio (index of γ-glutamyl cysteine synthetase activity a marker of glutathione synthesis) after 5, 10, and 21 days of bed rest. Data from the present studies (MARS-PRE 2019 and X-BRAIN-net 2023 bed rest studies) were combined with results obtained in different bed rest studies [44,45]. *, *p* < 0.05 bed rest vs ambulatory paired *t*-test with Bonferroni correction; #, *p* < 0.05 vs bed rest 5 days unpaired *t*-test with Bonferroni correction.

Figure 3 shows percent changes in glutathione concentration, γ-glutamyl cyclotransferase activity, and γ-glutamyl cysteine synthetase activity after 5, 10, and 21 days of bed rest. Data from the present studies (MARS-PRE 2019 and X-BRAIN-net 2023 bed rest studies) were combined with results obtained in different bed rest studies [44,45]. Glutathione concentrations decreased early to return to approximately baseline levels in the following days. Cysteinyl glycine-to-glutathione ratio, index of γ-glutamyl cyclotransferase activity, and a marker of glutathione degradation increased early to return to approximately baseline levels in the following days. γ-glutamyl cysteine-to-cysteine ratio, index of γ-glutamyl cysteine synthetase activity, and a marker of glutathione synthesis tended to increase throughout the bed rest period.

We compared glutathione kinetics, as determined by assessing the γ-glutamyl cycle, with the results of two previous studies [44,45] in which we measured glutathione turnover through the direct incorporation of L-[^2^H_2_-glycine] into the tripeptide. In one study [45], alkali supplementation significantly increased glutathione concentration by 5 ± 1% and decreased turnover by −37 ± 13%, which suggests a predominant inhibition of degradation rather than stimulation of glutathione synthesis. As confirmation of this kinetic assessment, the analysis of the γ-glutamyl cycle in stored blood samples indicated that alkali supplementation significantly (*p* < 0.05) decreased the cysteinyl-glycine-to-glutathione % ratio from 0.61 ± 0.06 to 0.54 ± 0.04, suggesting decreased γ-glutamyl cyclotransferase activity a marker of glutathione degradation. Alkali supplementation did not change significantly γ-glutamyl cysteine-to-cysteine % ratio from 4.61 ± 1.00 to 5.06 ± 1.00, a marker of glutathione synthesis and of the enzyme γ-glutamyl cysteine synthetase.

**Figure 4:**
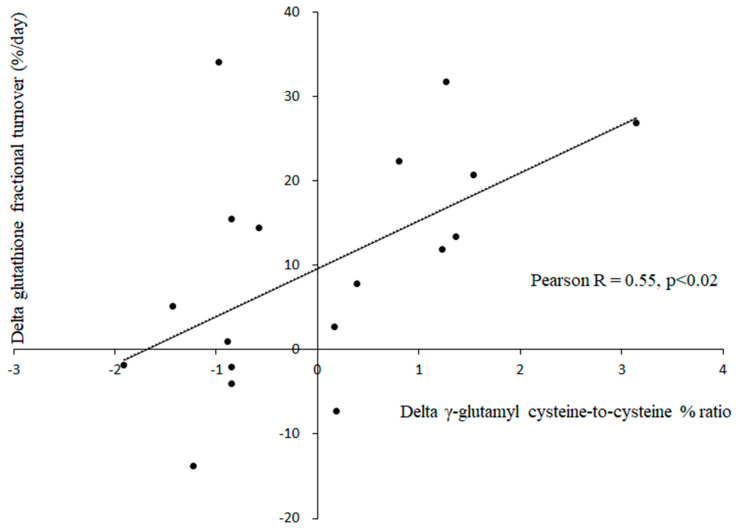
Relationships between hypoxia-mediated changes of γ-glutamyl cysteine-to-cysteine ratio and glutathione turnover in red blood cells, see Ref. [44].

The other study [44] investigated the effects of 10 days of experimental hypoxia in ambulatory or bed rest conditions on hematocrit, whole blood glutathione concentration, and RBC glutathione turnover, as shown in Table 5. Hypoxia significantly increased the hematocrit, glutathione concentration, and turnover, which suggests a predominant stimulation of synthesis rather than an inhibition of glutathione degradation. As confirmation of this kinetic assessment, γ-glutamyl cysteine synthetase significantly increased following hypoxia, while γ-glutamyl cyclotransferase did not change. Figure 4 shows the relationships (Pearson R = 0.55, *p* < 0.02) between hypoxia-mediated RBC changes of γ-glutamyl cysteine synthetase and glutathione turnover in ambulatory and bed resting subjects.

## 4. Discussion

In this study, we examined the changes in erythrocyte glutathione levels during a short-term bed rest experiment. By the 5th day of bed rest, we observed a significant reduction in glutathione levels, with concentrations dropping by approximately 13% from baseline. However, in the days that followed, glutathione levels rose significantly, returning to baseline by the 10th day of bed rest. Additionally, we measured the erythrocyte levels of intermediate metabolites in the γ-glutamyl cycle, a series of cytoplasmic reactions critical for glutathione synthesis and degradation. Each enzyme’s activity was assessed by calculating the ratio of product to precursor. Our findings showed that the initial depletion of glutathione during the first days of bed rest was linked to a significant increase in the cysteinyl-glycine-to-glutathione ratio and a trend toward an increase in the 5-oxoproline-to-glutathione ratio. These ratios, which reflect products of glutathione catabolism, indicate the activation of γ-glutamyl cyclotransferase, the main enzyme responsible for glutathione breakdown [24,25,26]. This early surge in glutathione degradation subsided in the following days. The importance of glutathione degradation in the initial decrease followed by the subsequent increase in glutathione levels is suggested by the inverse correlation between changes in the cysteinyl-glycine-to-glutathione ratio (i.e., γ-glutamyl cyclotransferase activity) and total glutathione concentration (*p* ≤ 0.01). Moreover, the rise in glutathione levels from the 5th to the 10th day was associated with significant increases in the γ-glutamyl cysteine-to-cysteine and γ-glutamyl cysteine-to-glutamate ratios, reflecting the activity of γ-glutamyl cysteine synthetase, a key regulator of glutathione synthesis [19,21].

The initial reduction in glutathione, likely driven by an increase in its catabolism, seems to be a response to oxidative stress during the early phase of bed rest. This inactivity-induced early oxidative stress is associated with both circulating [6] and muscle markers [7] of redox imbalance. In experimental models of muscle inactivity, including those in animals and humans, mitochondrial release of reactive oxygen species (ROS) has been observed, leading to the activation of antioxidant defenses [1]. Our previous research demonstrated that the early activation of oxidative stress markers in muscle, such as heme oxygenase-1 and mitochondrial heat shock protein-70, occurs before muscle atrophy becomes apparent, typically around the eighth day of short-term bed rest. During prolonged bed rest, oxidative stress markers return to baseline by day 35 [13].

Transient glutathione depletion is also seen in other clinical conditions characterized by acute oxidative stress, such as sepsis [48], experimental lipopolysaccharide administration [49], intense physical exercise [50], alcohol consumption [51], intermittent hypoxia in obstructive sleep apnea syndrome [52], hyperglycemia [53], and hyperinsulinemia [49]. Extensive evidence suggests that reduced glutathione levels are linked to oxidative stress, insulin resistance, endothelial dysfunction, muscle weakness, cardiac dysfunction [54,55], and non-alcoholic fatty liver disease (NAFLD) [56,57]. This supports the hypothesis that early glutathione depletion may contribute to the physiological changes commonly associated with physical inactivity, such as insulin resistance, dyslipidemia, muscle atrophy, and reduced muscle strength.

In this study, we assessed RBC glutathione kinetics using a novel method that measures intermediate metabolites of the γ-glutamyl cycle in erythrocytes. This five-enzyme cycle is essential for both the synthesis and degradation of glutathione. The clinical importance of the γ-glutamyl cycle in maintaining glutathione homeostasis is highlighted by the severe consequences observed in individuals with congenital enzyme defects [58]. γ-glutamylcysteine synthetase, the rate-limiting enzyme in glutathione synthesis, is critical in this process, with deficiencies leading to significant intracellular glutathione depletion and associated conditions like hemolytic anemia and neurological deficits [59]. Conversely, congenital disabilities in glutathione-degrading enzymes result in elevated intracellular concentrations and urinary excretion of the tripeptide, along with moderate intellectual disability [60]. Conditions such as oxoprolinuria, characterized by elevated 5-oxoproline levels, point to enzymatic deficiencies that impair glutathione synthesis, resulting from issues with enzymes like 5-oxoprolinase, glutathione synthetase, or γ-glutamylcysteine synthetase [61,62]. These deficiencies underscore the delicate balance needed to maintain appropriate glutathione levels and the potential systemic effects of disruptions in the γ-glutamyl cycle.

We validated the assessment of the cysteinyl-glycine-to-glutathione ratio (reflecting γ-glutamyl cyclotransferase activity) and the γ-glutamyl cysteine-to-cysteine ratio (indicating γ-glutamylcysteine synthetase activity) as indices of glutathione degradation and synthesis rates, respectively. This validation was conducted using blood samples from two previous studies [44,45], where erythrocyte glutathione kinetics were directly assessed by infusing a stable isotope of glycine and measuring its incorporation into the tripeptide. This method accurately evaluates glutathione turnover by simultaneously determining synthesis and degradation rates. However, changes in glutathione turnover should be interpreted in the context of tripeptide concentrations. When concentrations are stable, synthesis and degradation rates are balanced. An increase in concentration, along with a rise in turnover, suggests that synthesis is stimulated and surpasses degradation. Conversely, if turnover decreases while concentrations rise, degradation is inhibited without significant stimulation of synthesis.

In one study [45], administering an alkalizing agent to healthy volunteers during 21 days of bed rest led to decreased glutathione turnover and increased concentrations, attributed to reduced degradation. Retrospective analysis of blood samples from this study showed that alkalinization significantly increased γ-glutamyl cyclotransferase activity (measured by the cysteinyl-glycine-to-glutathione ratio) without affecting γ-glutamylcysteine synthetase activity (measured by the γ-glutamylcysteine-to-cysteine ratio). This finding confirms that the cysteinyl-glycine-to-glutathione ratio is a reliable marker of glutathione degradation. In the other study [44], healthy volunteers stayed in a hypoxic environment for 10 days under ambulatory and bed rest conditions, simulating an altitude of 4000 m. Hypoxia increased glutathione turnover and concentrations, attributed to enhanced synthesis. Retrospective analysis of blood samples from this study indicated that hypoxia significantly increased γ-glutamylcysteine synthetase activity (as shown by the γ-glutamylcysteine-to-cysteine % ratio) without affecting γ-glutamyl cyclotransferase activity (measured by the cysteinyl-glycine-to-glutathione % ratio). Additionally, hypoxia-induced changes in γ-glutamylcysteine synthetase activity were significantly correlated with changes in glutathione turnover (Figure 3). These results demonstrate that the γ-glutamylcysteine-to-cysteine % ratio serves as an indicator of the rate of glutathione synthesis. Therefore, modulating the activities of key enzymes in the γ-glutamyl cycle can effectively alter glutathione levels in response to different pharmacological and environmental conditions.

In a subgroup of 10 subjects, we determined the expression levels of the γ-glutamylcysteine synthetase enzyme in erythrocytes using Western blot analysis (Table 4). Despite bed rest-induced increases in γ-glutamylcysteine synthetase activity, based on increased product-to-precursor ratios, we observed a significant decrease in the expression of the modulator subunit of this enzyme, with no change in the catalytic subunit. This apparent discrepancy might be explained by a mechanism of metabolic feedback inhibition. High concentrations of γ-glutamylcysteine relative to cysteine could signal sufficient levels of the glutathione intermediate precursor, triggering a feedback mechanism that downregulates the modulator subunit of γ-glutamylcysteine synthetase to prevent overproduction. This view is supported by the inverse correlation between bed rest-induced changes in the γ-glutamylcysteine-to-cysteine ratio from baseline to the fifth day and changes in the modulator subunit of γ-glutamylcysteine synthetase (R = −0.70). Evidence suggests that cysteine supplementation is linked to increased glutathione synthesis [23,55,63]. Thus, cysteine may directly decrease the γ-glutamylcysteine-to-cysteine ratio, leading to upregulation of the modulator subunit of γ-glutamylcysteine synthetase.

We integrated data from the recent studies MARS-PRE 2019 and X-BRAIN-net 2023 with information from earlier bed rest studies [44,45] to assess how bed rest duration, up to 21 days, affects erythrocyte glutathione levels. We examined these changes in relation to the processes of glutathione synthesis and degradation. Glutathione concentrations initially dropped by day 5 but returned to near baseline levels by day 10 and were sustained through day 21. The activity of γ-glutamyl cyclotransferase, an indicator of glutathione degradation, also increased early on but reverted to baseline levels afterward. In contrast, γ-glutamylcysteine synthetase activity, a marker of glutathione synthesis, tended to rise throughout the bed rest period.

Fluctuations in glutathione concentration arise from variations in its synthesis and degradation rates. We have developed and validated a novel technique for measuring intermediates of the γ-glutamyl cycle in erythrocytes, which enables simultaneous assessment of glutathione synthesis and degradation rates. This method helps pinpoint the underlying causes of changes in glutathione kinetics, especially in conditions that impact redox balance and intracellular glutathione levels. Nutritional interventions, such as cysteine, glycine, selenium, and curcumin supplementation, can enhance intracellular glutathione levels by promoting its synthesis or potentially inhibiting its degradation [23,29,55,63,64,65].

## 5. Study Limitations

While this study provides valuable insights into the role of the γ-glutamyl cycle in glutathione turnover during short-term bed rest, it has some limitations. First, the sample size was relatively small, consisting of only 19 healthy young male volunteers, which limits the generalizability of the findings to other populations, such as females, older adults, or individuals with underlying health conditions. Another limitation is that glutathione concentrations were measured only in erythrocytes, which may not accurately reflect glutathione kinetics in other tissues, such as muscle or liver, where oxidative stress and glutathione turnover may behave differently. Future studies should aim to include a larger, more diverse sample and assess glutathione kinetics across multiple tissues over a longer period to provide a more comprehensive understanding of the effects of physical inactivity.

## 6. Conclusions

In summary, we have created a simple, non-invasive method to evaluate intracellular glutathione kinetics by measuring intra-erythrocytic γ-glutamyl cycle intermediates. This technique allows for the detailed characterization of glutathione depletion conditions, differentiating between accelerated degradation and reduced synthesis. It also facilitates targeted pharmacological and nutritional interventions to boost glutathione levels or prevent its depletion and enables the assessment of the effectiveness of these interventions.

## Figures and Tables

**Table 1:** Bed rest effects on plasma concentrations of basic biochemical parameters and markers of insulin sensitivity and secretion.

	Baseline	5th BR Day	10th BR Day	*p* BR Effect
Insulin (µU/mL)	6.4 ± 2.7	7.3 ± 3.6	8.1 ± 4.1	0.041
Glucose (mg/dL)	86 ± 4	84 ± 3	82 ± 5	0.004
HOMA-IR index	1.3 ± 0.6	1.5 ± 0.7	1.7 ± 0.9	0.096
HOMA-β%	104 ± 50	129 ± 66	159 ± 84	0.006
Total cholesterol (mg/dL)	144 ± 31	141 ± 24	136 ± 23	0.013
LDL cholesterol (mg/dL)	85 ± 27	85 ± 20	81 ± 19	0.048
HDL cholesterol (mg/dL)	43 ± 10	40 ± 11	39 ± 10	0.001
Triglyceride (mg/dL)	76 ± 20	83 ± 26	81 ± 26	0.412
Triglyceride/HDL cholesterol	2.0 ± 1.0	2.3 ± 1.1	2.3 ± 1.1	0.011
ALT (mU/mL)	19 ± 7	19 ± 7	23 ± 12	0.182
AST (mU/mL)	36 ± 8	37 ± 7	39 ± 8	0.267
γGT (mU/mL)	18 ± 1	19 ± 2	21 ± 3	0.807
CRP (mg/L)	0.8 ± 1.1	0.6 ± 1.2	0.6 ± 0.8	0.439
Creatinine (mg/dL)	1.0 ± 0.1	1.1 ± 0.1	1.1 ± 0.1	0.154

BR: Bed Rest; HOMA-IR: homeostatic model assessment—insulin resistance; HOMA-β%: homeostatic model assessment—insulin secretion (β-cell function); LDL: low-density lipoprotein; HDL: high-density lipoprotein; ALT: alanine transaminase; AST aspartate transaminase; γGT: γglutamyl transferase; CRP: C-reactive protein.

**Table 2:** Bed rest effects on the ratio between reduced and oxidized glutathione as well as the levels of total glutathione and of intermediate substrates of the γ-glutamyl cycle in erythrocytes.

	Baseline	5th BR Day	10th BR Day	*p* BR Effect
Total glutathione	2268 ± 619	2093 ± 689 *	2279 ± 671 ^#^	<0.001
GSH/GSSG	247 ± 70	291 ± 87	184 ± 18	0.34
γ-glutamyl alanine	73 ± 28	70 ± 27	78 ± 30	0.223
Cysteinyl-glycine	9.3 ± 1.0	9.5 ± 0.7	9.3 ± 1.5	0.686
5-oxoproline	199 ± 36	183 ± 34	187 ± 29	0.263
Glutamic acid	285 ± 72	280 ± 67	275 ± 68	0.548
γ-glutamyl cysteine	3.2 ± 1.2	3.4 ± 1.2	3.2 ± 1.0	0.603
Glutamine	451 ± 35	459 ± 34	438 ± 47	0.120
Cysteine	103 ± 30	101 ± 31	90 ± 19	0.020
Glycine	470 ± 102	476 ± 112	478 ± 104	0.671
Alanine	258 ± 38	288 ± 35	298 ± 36	0.001

BR: Bed Rest; GSH/GSSG: the ratio between reduced and oxidized glutathione. Values are presented as mean ± SD. *p*, repeated measures ANOVA or ANCOVA, post hoc analysis by paired *t*-test with Bonferroni correction; *, *p* ≤ 0.05 versus baseline; ^#^, *p* ≤ 0.05 versus 5th bed rest day. *N* = 19. Units are nmol/mL erythrocyte volume except for the GSH/GSSG ratio. Concentrations of total glutathione and of intermediate substrates of the γ-glutamyl cycle in erythrocytes were determined using gas chromatography-mass spectrometry and stable isotopes as internal standards. The ratio between reduced and oxidized glutathione was directly determined by the enzymatic method. See Section 2.2.

**Table 3:** Bed rest effects on γ-glutamyl cycle enzyme activities estimated as product-to-precursor ratios in erythrocytes.

	Baseline	5th BR Day	10th BR Day	*p* BR Effect
Cysteinyl-glycine/glutathione, % (γ-GCT)	0.43 ± 0.09	0.49 ± 0.13 *	0.43 ± 0.10 ^#^	0.013
5-oxoproline/glutathione, % (γ-GCT)	9.25 ± 2.44	9.59 ± 3.28	8.96 ± 2.94	0.340
glycine/cysteinyl-glycine, ratio (CGD)	50 ± 9	50 ± 9	53 ± 16	0.422
cysteine/cysteinyl-glycine, ratio (CGD)	11.2 ± 3.9	10.6 ± 2.7	9.9 ± 2.8	0.030
glutamate/5-oxoproline, ratio (OA)	1.4 ± 0.3	1.6 ± 0.4	1.5 ± 0.4	0.029
γ-glutamyl cysteine/cysteine, % (γ-GCS)	3.12 ± 0.81	3.41 ± 0.97	3.61 ± 0.62	0.015
γ-glutamyl cysteine/glutamate, % (γ-GCS)	1.14 ± 0.36	1.21 ± 0.33	1.18 ± 0.34	0.006
Glutathione/γ-glutamyl cysteine, ratio (GS)	769 ± 244	646 ± 156	757 ± 234	0.17
Glutathione/glycine, ratio (GS)	4.8 ± 0.8	4.4 ± 0.8	4.7 ± 0.8	0.07

BR, Bed Rest; γ-GCT, γ-glutamyl cyclotransferase; CGD, cysteinyl glycine dipeptidase; OA, 5-oxoprolinase; γ-GCS, γ-glutamyl cysteine synthetase; GS, glutathione synthetase. Values are presented as mean ± SD. *p*, repeated measures ANOVA or ANCOVA, post hoc analysis by paired *t*-test with Bonferroni correction; *, *p* ≤ 0.05 versus baseline; ^#^, *p* ≤ 0.05 versus 5th bed rest day. *n* = 19.

**Table 4:** Bed rest effects on expression levels of the γ-glutamyl cysteine synthetase enzyme in erythrocytes.

	Baseline	5th BR Day	10th BR Day	*p* BR Effect
γ-GCS-C (fraction of GAPDH)	0.54 ± 0.13	0.54 ± 0.16	0.54 ± 0.16	0.773
γ-GCS-M (fraction of GAPDH)	1.00 ± 0.16	0.95 ± 0.09	0.96 ± 0.12	0.003
γ-GCS-M/γ-GCS-C (ratio)	1.98 ± 0.61	1.89 ± 0.58	1.95 ± 0.66	0.512

BR, Bed Rest; γ-GCS-C, γ-glutamyl cysteine synthetase—catalytic subunit; GAPDH, glyceraldehyde-3-phosphate dehydrogenase; γ-GCS-M, γ-glutamyl cysteine synthetase—modulator subunit. *p*, repeated measures ANOVA or ANCOVA, *n* = 10.

**Table 5:** The effects of hypoxia in ambulatory and bed rest conditions on glutathione concentrations, fractional turnover rate, and markers of glutathione degradation and synthesis in erythrocytes; see Ref. [44].

	Ambulatory		10-Day Bed Rest		*p* *		
	Normoxia	Hypoxia	Normoxia	Hypoxia	Bed Rest Effect	Hypoxia Effect	Interaction
Hematocrit (%)	46 ± 1	47 ± 1	51 ± 1	51 ± 1	0.20	<0.001	0.12
Glutathione concentration (mmol/L WB)	1245 ± 49	1331 ± 52	1236 ± 54	1287 ± 51	0.22	0.01	0.45
Glutathione Fractional Turnover (%/day)	25 ± 4	38 ± 6	35 ± 3	41 ± 3	0.09	0.03	0.21
^a^ Cysteinyl-glycine/glutathione, % ratio	0.33 ± 0.01	0.36 ± 0.02	0.36 ± 0.02	0.35 ± 0.01	0.08	0.10	0.53
^b^ γ-glutamyl cysteine/cysteine, % ratio	4.342 ± 0.341	4.344 ± 0.291	4.238 ± 0.241	4.280 ± 0.258	0.06	0.03	0.14

WB, whole blood. *, data were analyzed using a 2-factor repeated measure ANOVA with interaction. n = 11. ^a^, marker of glutathione degradation and of the enzyme γ-glutamyl cyclotransferase. ^b^, marker of glutathione synthesis and of the enzyme γ-glutamyl cysteine synthetase.

## Data Availability

The data included in this study are available from the corresponding authors upon reasonable request.
